# The Effect of Specific Strength Training on Throwing Velocity in Overarm Throwing: A Systematic Review

**DOI:** 10.1186/s40798-024-00785-7

**Published:** 2024-11-09

**Authors:** Andrea Bao Fredriksen, Roland van den Tillaar

**Affiliations:** Department of Sports Sciences and Physical Education, Nord University, Levanger, Norway

**Keywords:** Overweighted Balls, Underweighted Balls, Pulley Device, Wearable Resistance

## Abstract

**Background:**

Throwing velocity is an important research topic in sports science, and it is an essential performance variable for athletes in overarm-throwing sports like baseball, team handball, water polo, softball, and cricket. The aim of the present review was to investigate the effect of specific strength training on throwing velocity for overarm throws.

**Methods:**

The literature was analysed using the Preferred Reporting Items for Systematic reviews and Meta-analyses, searching in SPORTDiscus and MEDLINE. Seventeen studies were included in this review, and the training studies were divided into four categories: (a) overweight and underweight balls, (b) forearm loading, (c) pulley device training, and (d) strength training with a resistance band.

**Results:**

All strength training studies with resistance band and the forearm loading categories increased the throwing velocity, varying from 3.7 to 26%. However, only half of these studies found that training was associated with a significantly higher increase versus the control group. Findings were inconsistent in other categories.

**Conclusion:**

Based on the findings of the present review, no clear conclusion can be made on which of the specific strength training methods is best for increasing throwing velocity. However, some recommendations can be offered. Firstly, the throwing training period should be long enough (≥ 6 weeks) with a high enough workload. Throwing training with a resistance band increases throwing velocity significantly for junior and less experienced overarm-throwing athletes. Furthermore, throwing with underweighted balls of similar size will ensure a positive effect on throwing velocity. Also, throwing training with combined over- and underweighted balls can be used if the overweighted balls are carefully selected to ensure there is no negative impact on throwing kinematics and injuries. For the other categories, the results were conflicting. Furthermore, due to the low number of studies in the pulley device and forearm loading categories, more studies should be conducted to investigate their effects on throwing velocity.

## Key Points

• All strength training studies with resistance band and the forearm loading categories increased the throwing velocity, varying from 3.7 to 26%.

• Only half of these studies found that training was associated with a significantly higher increase versus the control group, while findings were inconsistent in other categories.

• Due to the low number of studies in the pulley device and forearm loading categories, more studies should be conducted to investigate their effects on throwing velocity.

## Background

Overarm throwing is an important technique in many different sports (e.g., team handball, water polo, baseball, softball, and cricket) because it is an essential performance variable for an overarm-throwing athlete that could predict success or failure in overarm-throwing sports [1–3]. There are several training methods that aim to increase the throwing performance in these overarm-throwing sports. In strength training, we often differentiate between general strength training and specific strength training [4]. General strength refers to the training on the major muscle groups and the whole muscular system, and specific strength training relates to the movements in a specific sport. To achieve the most specific training possible, the movement pattern in training should reflect the movement pattern in competition. This has been in focus for some time and is important in training for performance [5]. The most specific training for overarm throwing is practicing with the same technique and movement pattern as in the sports.

Earlier reviews have attempted to summarise the effects of different training methods on improving throwing velocity [2, 3, 6–8]. These reviews concluded that several training methods (e.g., general strength training, resistance band training, throwing with over- and underweight balls) can be effective to increase throwing velocity. However, other factors like age, sex, training experience, and fatigue must be taken into consideration. Previous reviews have examined the impact of different training methods, such as general strength training and specific strength training [1, 9–12]. However, none of these studies has solely focused on investigating the effectiveness of specific strength training or providing explanations about the underlying mechanisms of these training techniques.

Only van den Tillaar [3] included specific strength training methods for overarm throwing (e.g., over- and underweight balls) in his review. He categorised strength training interventions by two primary overload principles: the principles of specificity and overload that can be used to increase throwing velocity. Thereby, he classified specific strength training with overload of velocity as throwing with underweight balls, while specific strength training with overweight balls followed the principle of force. The main findings in his review were that the use of lighter balls always enhanced throwing performance with the normal balls, while the use of overweight balls gave conflicting results. That review was conducted almost 20 years ago and only included specific strength training with under- and overweight balls or a combination of both. In that review, no studies were included using elastic bands or other newly developed equipment like wearable resistance on the upper arm. It is expected that new studies on specific strength training for overarm throwing after the time of the review have been conducted.

Therefore, the aim of the present review was to investigate the training effect on throwing velocity of specific strength training for overarm throw. Since there have been several newer reviews on the long-term effect of general strength training upon throwing performance [1, 3, 6–8, 13], general strength training studies were excluded in this review.

## Methods

### Experimental Approach to the Problem

The literature was collected by searching the SPORTDiscus and MEDLINE databases on the 1th of October 2023. The specific terms in these databases utilised: (strength training OR resistance training OR Overload training OR wearable resistance OR specific strength training) AND (overarm throw OR throwing OR underweight balls OR wearable resistance OR specific strength training OR overarm throw OR throwing OR underweighted balls OR elastic band training OR throwing velocity). Combinations of these words were sometimes used to limit the number of articles to review. These studies were then narrowed down by title according to relevance to throwing and/or specific strength measures for throwing.

### Inclusion Criteria

**Fig. 1:**
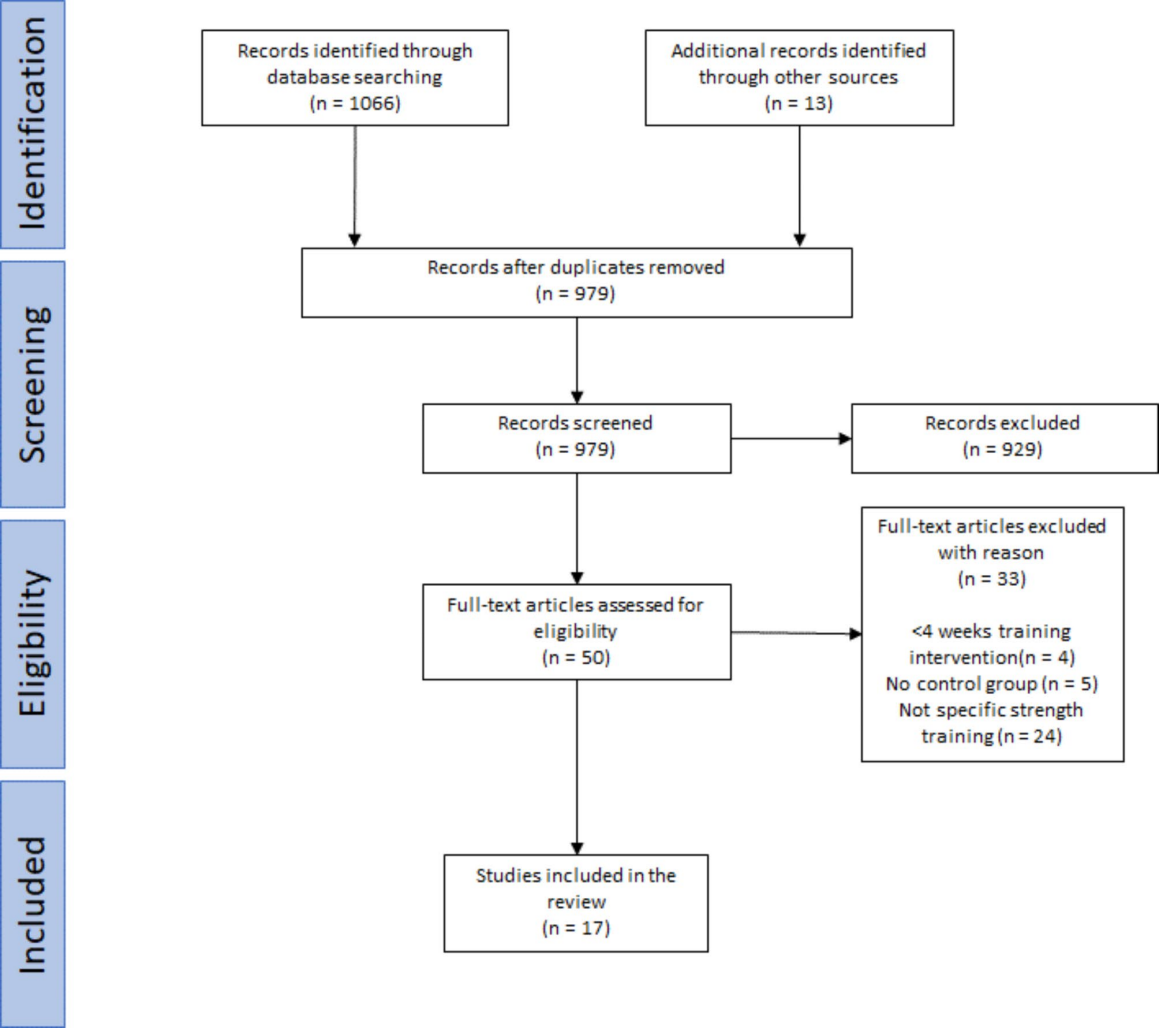
PRISMA flowchart showing application of inclusion and exclusion criteria to search results

Studies included in the review were required to (a) be published in English, (b) be peer reviewed, (c) include an intervention with a minimum of 4 weeks of training, (d) test the effect of the training intervention on throwing velocity, (e) conduct specific strength training for overarm throw with one hand, (f) measure throwing velocity, and (g) be published after 2003, since the brief review by van den Tillaar [3] has already discussed the findings from specific strength training studies from before 2003. Articles with one or more of the following criteria were excluded: (a) reviews, (b) overhead throw, (c) cross-sectional studies, (d) no control group (e) master theses. A representative search strategy is shown in Fig. 1, using Preferred Reporting Items for Systematic reviews and Meta-analyses (PRISMA) guidelines [14]. The searches were performed by one researcher.

## Results

Seventeen papers on specific strength training for overarm throwing met the inclusion criteria and were reviewed. The papers were divided into four categories according to the principles of training and the different influence of the load: (a) overweight and underweight balls, which gives overload of velocity and overload of strength when throwing the ball; (b) forearm loading, which gives overload in strength for a longer period, also when the ball is thrown away relative to overweighted balls; (c) pulley device training, which gives an overload in strength with pulling constant resistance; (d) strength training with a resistance band, which gives a small load at the beginning of the exercise and increases in load at the end of the movement. Ekaterini et al. [15] had a light ball training group and a forearm loading group, which were in different categories. Therefore, these are mentioned in both categories with their training and results compared to the control group.

Of the 17 studies, 12 involved handball, two cricket and baseball, and one softball (Table 1). Four of the 17 studies involved women, while the rest included only men. The shortest intervention period was only 4 weeks [16]; on average, the training period was 6–8 weeks, while five studies had the longest intervention period of 10 weeks [15, 17–20]. Most studies included experienced junior or senior players; however, three studies included students who were novices in their sport [15, 17, 19]. Nine studies [15, 16, 18–24] investigated the effects of over- and underweighted balls (Table 1). In the forearm loading and pulley device categories, two studies each (Table 2) were performed [15, 17, 25, 26], while in the elastic resistance category, five studies (Table 3) were included [27–31].

### Training with Over- and Underweighted Balls

When specified per category, this category can be divided into three different sub-categories: training with underweighted or overweighted balls or a combination of both. Four studies were found that only trained with underweighted balls [15, 16, 19, 20], four that used a combination [18, 22–24], and only one that used overweighted balls [21]. The control groups in all the over- and underweighted ball studies continued with normal training (throws with the standard weighted balls in their respective sports). Some studies controlled that the experimental group and the control group had the same workload [21, 23], and the workload for these studies was calculated with the impulse (∫Fdt) generated per throwing attempt [26]. Other studies controlled that the control group (with standard balls) had the same number of throws as the other groups [16, 18, 20, 22], while Reinold et al. [24] only reported that the control group should continue with normal training and not train with under- or overweighted balls in the experimental period.

**Fig. 2:**
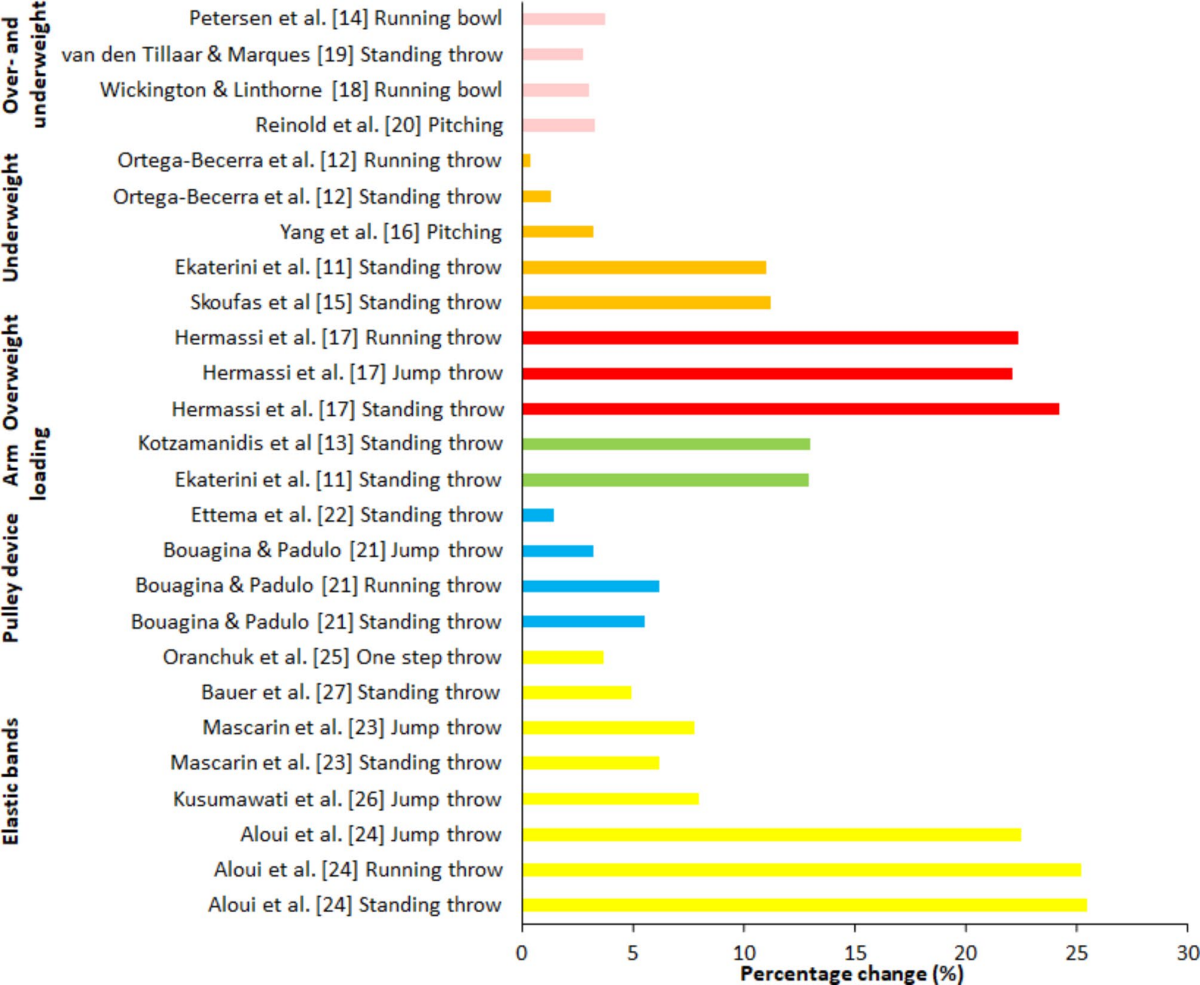
Percentage of change from pre- to post-test for the different intervention groups

Six of the nine studies reported a significant increase in throwing velocity (Table 1; Fig. 2) within the intervention group of 3–24.2% [15, 19–22, 24]. Some studies used several training programmes and a control group to determine if one type of training increased throwing velocity more than the other. Thereby, comparing the change in velocity with the control groups, only three studies — two in the underweighted sub-category [19, 20] and one in the overweighted sub-category [21] — showed a significantly higher increase in velocity in the intervention group compared with the control group.

**Table 1:** Effect of training with overweight and underweight balls on throwing velocity per intervention and control group

Reference	Sport	Sex	*n*	Level	Duration (week)	Training intervention group (absolute increase)	Significant (%)	Control group (absolute increase)	Significant (%)	Between groups (%)
**Overweight and underweight balls**										
Petersen et al. [19]	Cricket	M	16	Senior	10	Modified-implement training: underweight 161–181 g, overweight 151–131 g, and normal 156 g (+ 1.1 m/s)	No (3.7)	Normal training (+ 0.4 m/s)	No	No
van den Tillaar and Marques [24]	Handball	F	20	Senior	8	Variable training with under- (0.288 kg) and over-weight balls (0.432 kg) (+ 0.5 m/s)	No (2.8)	Normal training	No	No
Wickington and Linthorne [23]	Cricket	M	8	Senior	8	Weighted balls: 71, 113, 142, 198, and 213 g (+ 1.0 m/s)	Yes (3)	Standard weight balls (+ 0.2 m/s)	No	No
Reinold et al. [25]	Baseball	M	34	13–18 years	6	Weighted balls: 2–32 ounces (+ 1.0 m/s)	Yes (3.3)	Normal training (+ 0.3 m/s)	No	No
**Underweight balls**										
Ortega-Becerra et al. [17]	Handball	M	24	Senior	4	1. Tennis ball (+ 0.3 m/s) 2. Medicine ball non-specific throw (-0.2 m/s)	No	Same number of throws (+ 0.3 m/s)	No	No
Yang et al. [21]	Baseball	M	24	Junior	10	Lightweight baseball training: 4.4 ounces (+ 0.9 m/s)	Yes (3.2)	Normal training (+ 0.2 m/s)	No	Yes (2.4)
Ekaterini et al. [16]	Handball	M	65	College novice	10	Training with 20% lighter balls (+ 2.0 m/s)	Yes (11.0)	Same number of throws (+ 1.1 m/s)	No (6.1)	No
Skoufas et al. [20]	Handball	M	43	College novice	10	Training with 20% lighter balls (+ 2.0 m/s)	Yes (11.2)	Same number of throws (+ 1.2 m/s)	No (6.5)	Yes
**Overweight balls**										
Hermassi et al. [22]	Handball	M	34	Elite	8	1. Resistance training group, throwing with 3-kg medicine ball (standing throw + 4.7 m/s, jump throw + 5.1 m/s, and running throw + 5.7 m/s) 2. Regular training group, throwing with regular handballs, size 3 (jump throw + 3.9 m/s)	Yes (16.7–24.2)	Normal training	No	Yes

M = men, F = women, n = number of participants

Hermassi et al. [21] found that training with a medicine ball (overweight) did improve throwing velocity more than throwing with regular balls (handball balls) and the control group, which did not perform extra throwing training. From pre- to post-test in all three conditions (jump throw, running throw, and standing throw), the velocity increased significantly in the medicine ball group (22–24%), which was matched in throwing workload with the regular ball training group, in which there was a significant increase only in jump throw (+ 16.7%). However, van den Tillaar and Marques [23] also matched throwing workload between the under- and overweighted training group with the regular balls training group without any significant differences within and between groups (Table 1).

### Throwing Training with Forearm Loading and Pulley Device

The two forearm loading studies [15, 17] had similar training groups: college students who were novices in handball, with the same kind of training programme and similar increases in ball velocity in both the intervention (12.9–13%) and control groups (6.1–6.3%). The two studies with the pulley device with the same intervention period of 8 weeks [25, 26] showed different outcomes: Ettema et al. [26] did not find any positive effects (+ 1.4%) in female senior handball players, whereas Bouagina et al. [25] with junior male handball players found significant improvements after the intervention of 3.2 (jump throw) to 6.2 (running throw) whereas the control group did not increase throwing performance (Table 2; Fig. 2).

### Throwing Training with a Resistance band

The five studies [27–31] that used resistance (elastic) bands all included handball players, except for the study by Oranchuk et al. [29], who included softball players. Furthermore, some studies focused on shoulder strengthening [29, 31], while others focused more on the forearm [30] and throwing joint movements [27, 28]. All the studies reported increases in throwing velocity after the intervention period, varying from 3.7% [29] to 26.1% [28], with three of the five studies showing significantly higher increases after resistance band training (Table 3) compared with the control group [28, 30, 31].

When investigating the percentages of change after the training periods for diverse throws that were studied in the categories, percentages varied from 1.4 to 26.1% (Fig. 2). Of the 18 interventions, four did not report significant increases after the intervention period. However, when compared to the control groups, only eight observed significantly higher increases [17, 19–21, 25, 28, 30, 31].

**Table 2:** Effect of different specific strength training methods (forearm loading and pulley device) on throwing velocity per intervention and control group

Reference	Sport	Sex	*n*	Level	Duration	Training intervention group	Significant	Control group	Significant		Between
					(week)	(absolute increase)	(%)	(absolute increase)	(%)		groups (%)
***Forearm loading***											
Kotzamanidis et al. [18]	Handball	M	41	College, novice	10	Training with + 107 g and + 84 g on the upper arm and forearm. Same torque over the shoulder, as a 20% heavier handball would induce (+ 2.3 m/s)	Yes (13.0)	Same number of throws (+ 1.1 m/s)		Yes (6.3)	Yes (5.3)
Ekaterini et al. [16]	Handball	M	65	College, novice	10	Training with + 107 g and + 84 g on the upper arm and forearm. Same torque over the shoulder, as a 20% heavier handball would induce (+ 2.3 m/s)	Yes (12.9)	Same number of throws (+ 1.1 m/s)		No (6.1)	No
***Pulley device***											
Ettema et al. [27]	Handball	F	13	Senior	8	Resistance training with a pulley device 85% of 1RM (+ 0.25 m/s)	No (1.4%)	Same throwing workload with regular ball (+ 1.0 m/s)		Yes (6.1%)	No
Bouagina et al. [26]	Handball	M	26	Junior	8	Handball specific movement patterns using an arm/shoulder specific strength device to mimic handball shot (standing throw: +1.18 m/s; running throw: +1.37 m/s; jump throw: +0.71 m/s)	Yes (ST: 5.55; RT: 6.21; JT: 3.19)	Normal training (ST: -0.04 m/s; RT: +0.12 m/s; JT: +0.35 m/s)		No	Yes (ST: 5.73; RT: 5.4; JT: 1.4)

M = men, W = women, n = number of participants, ST = standing throw, JT = Jump throw, RT = running Throw, 1RM = one repetition maximum

**Table 3:** Effect of different specific strength training with resistance bands on throwing velocity per intervention and control group

Reference	Sport	Sex	*N*	Level	Duration	Training intervention group	Significant	Control group	Significant	Between
					(week)	(absolute increase)	(%)	(absolute increase)	(%)	groups (%)
Oranchuk et al. [30]	Softball	F	28	College	8	Sport-specific resistance training programme with resistance bands (+ 0.88 m/s)	Yes (3.7)	General resistance training with resistance bands (+ 0.42 m/s)	No	No
Bauer et al. [32]	Handball	M	32	Junior	9	Shoulder strengthening programme with elastic bands (+ 1.05 m/s)	Yes (4.9)	Normal training (+ 0.11 m/s)	No	Yes
Mascarin et al. [28]	Handball	F	39	Junior	6	Progressive strength training programme with elastic bands (standing throw: +0.86 m/s; jump throw: +1.22 m/s)	Yes (ST: 6.2; JT: 7.8)	Normal training (ST: +0.61 m/s; JT: +0.69 m/s)	No	No
Kusumawati et al. [31]	Handball	M	28	University	8	Light resistance training programme using elastic bands and body weight as resistance for forearm training (+ 1.35 m/s)	Yes (8)	Normal training and no forearm resistance training (0.26 m/s)	No	Yes (6)
Aloui et al. [29]	Handball	M	30	Junior	8	Progressive strength training programme with elastic bands (standing throw: +5.4 m/s; running throw: +5.9 m/s; jump throw: +5.5 m/s)	Yes (25.1–26.1)	Normal training (ST: +0.9 m/s; RT: +0.9 m/s; JT: +1 m/s)	Yes (3.9–4.4)	Yes

M = men, W = women, n = number of participants, ST = standing throw, JT = Jump throw, RT = running Throw

## Discussion

The purpose of this review was to investigate the training effect on throwing velocity of specific strength training for overarm throwing and to represent a theoretical framework of the findings. The main findings in this review were that all studies in the strength training with resistance band category and the forearm loading studies increased throwing velocity, varying from 3.7 to 26%. However, when compared with the corresponding control groups, only half of these studies had a significantly higher increase. For the other categories, the findings were conflicting. Four studies found a significant increase in throwing velocity, and three studies did not with over- and underweighted balls. Two studies in the pulley device category also showed conflicting results, where Bouagina et al. [25] found a significant increase, and Ettema et al. [26] did not. Furthermore, the forearm loading studies gave no clear answers, because of the significant differences found for both groups.

When evaluating the improvements in the different studies, there are several possible explanations for the findings. One of the main reasons for improvement is probably neural adaptation in the under- and overweighted balls and resistance band studies, because neural adaptation is affected earlier and more quickly by training than a change in muscle fibre type, muscle growth or other muscle adaptations that are measurable over these training periods [32]. This was also one of the explanations for the findings in the previous review by van den Tillaar [3] on specific throwing training with under- and overweight balls. One of these neural adaptations is increased internal shoulder rotation, as found by van den Tillaar and Marques [23]. This movement is also identified as one of the main contributors to overarm throwing performance [33]. Another reason for improvements is increased strength of the involved muscles. Increased strength levels in pullover and bench press [21, 28], hand grip [30], maximal isometric internal shoulder rotation strength [31], 1-RM chop test [29], and average isokinetic concentric internal shoulder rotation power at 240°/s [27] were also found, indicating higher strength levels of the involved muscles for throwing. However, these increased strength levels did not always parallel the changes in throwing velocity. Oranchuk et al. [29] found increased 1-RM strength at around 23–26%, and throwing velocity increased by only 3.7% after the specific elastic band training, while general elastic band training only resulted in a 1.7% increase in throwing velocity, indicating that the strength training has to be specific. Whereas Ettema et al. [26] reported increases of 11.8% and 22.8% in weight when making a throwing movement at 85 and maximal 1-RM with a pulley device, an increase of only 1.4% in throwing with a regular ball was found, indicating that extra strength does not always result in faster throwing. The control group, which only trained with regular balls, had a higher increase of 6.1% [26]. Furthermore, Oranchuk et al. [29] found only non-significant small correlations between change in 1-RM strength test and peak velocity.

Another explanation for the enhanced throwing velocity after intervention is the increased external rotation range of motion, which gives the thrower a longer throwing trajectory in which force can be produced in the acceleration phase of the throw [34]. Reinold et al. [24] showed that training with different weighted balls resulted in an increased throwing velocity (3.3%) and external shoulder rotation (4.3%), which could be responsible for this increase. However, an overall injury rate of 24% also occurred in this training group, indicating that this type of training also could result in more injuries.

Other reasons for improvements in throwing velocity are learning effect, level, and experience of the athletes. The learning effect could also be seen as a shortcoming in some of the studies, as from pre- to post-test the players have learned the test procedure and thereby “learned” to throw faster. This could explain why both groups found an increase, as well as the lack of significant difference between groups [17, 28, 35]. Furthermore, novices have more potential to increase their throwing velocity by any sort of training due to the lack of throwing training compared to experts, which was shown in the forearm studies [15, 17] and underweight studies of Skoufas et al. [19] and Ekaterini et al. [15], who assessed novice handball players. This resulted in increases of 11–12%, while similar underweight training studies with experienced players [20, 36] resulted in increases of only 2–3%.

There are also improvements in throwing velocity that are doubtful. Hermassi et al. [37] and Aloui et al. [28] reported increases of 16.7–26.1% after training with reps. heavy medicine balls and elastic band training. These increases exceed throwing enhancements found in most studies (3–10%) on overarm throwing, as shown in the earlier review by van den Tillaar [3]. A reason for these large increases could be the inexperience of testing at pre-test. An indication for this is that the regular throwing training group in Hermassi et al. [37] study also showed an increase in throwing velocity in the jump shot of 16%.

There are also several explanations for the lack of improvement in throwing velocity. Some reasons for the lack of improvement in the reviewed studies could be the short intervention period, the level, and the age of the athletes. Ortega-Becerra et al. [16] did not find a significant increase in the under- and overweight balls category. The training intervention lasted for only 4 weeks, and they tested senior athletes. The better trained the athletes, the more difficult it is to make progress in training, especially over such short periods. This could also be an explanation for the lack of improvement in the studies by van den Tillaar and Marques [23] and Petersen et al. [18]; because of the high level of the athletes, it was harder to gain a positive effect of the training.

Furthermore, results are also influenced by the number of subjects tested, and the lack of significant difference could be because some of the studies did not test enough subjects [18, 22, 23, 26]. Ettema et al. [26] found an increase in the experimental group and the control group, but due to the low number of subjects in each group, the increase was not significant. However, when compared between groups, no significant difference was found in throwing velocity. Even when one group had a larger increase than the other due to variability (low participation in each group), the increase was not significant. When both groups were analysed together, the throwing velocity was significantly higher from pre- to post-test due to the higher number of subjects.

Another reason for the lack of significant differences between the groups could be fatigue in the experimental group [27]. It can be hypothesised that athletes’ muscles have a capacity limit for strength improvement, and the same training programme for weeks may cause fatigue, limiting the strength gain. However, too low a workload could also be an explanation for the lack of improvement. According to Schmidtbleicher [38], low loads do not overload the muscles enough to induce an adaptation. This was tested in general strength, but the same principles for strength adaptations apply for specific strength. However, the intensity and the specific technique are the most important variables in resistance training for highly trained athletes trying to improve throwing velocity. Therefore, studies may have found no progress [16, 18, 23, 26] because it is more difficult improving speed than strength. In these studies, the size principle of motor recruitment is a possible explanation for the lack of positive effect.

When evaluating the specific strength training per category, it was found that strength training with a resistance band was the only category in which all studies observed a significant increase in throwing velocity. An explanation for this could be the different loads given during the exercise, with a small load at the beginning and a larger load at the end. When training with a resistance band, the high-speed movements entail an important coordination component that is not trained in low speed/high resistance exercises when using a pulley device [26]. Thus, it can be assumed that the resistance band gave a high enough load to have a training effect. The results support that intensity may be a major factor for improving throwing velocity, which agrees with earlier findings [2, 3, 7].

In studies not using elastic bands, resistance from high-force and low-velocity pulley devices resulted in conflicting results. In the study by Ettema et al. [26], the control group improved more than the training group, which could be due to the importance of specificity. The lack of improvement in throwing velocity could be explained by the lack of adaptation in coordination dynamics, which may be a more important factor than increase in strength [26]. On the other hand, Bouagina et al. [25] found a significant increase in throwing velocity using an arm/shoulder specific strength device. Comparison of these studies is difficult because of the different study designs, measurements methods, skill levels, ages, and sexes of the participants and the intensity of the training. Bouagina et al. [25] tested male junior handball players, while Ettema et al. [26] tested female senior players, and less experienced players adapt more easily to training than high-performing senior athletes. However, only two studies were found in this category, which makes it difficult to state whether this type of training works for enhancing throwing performance.

In the categories of over- and underweighted balls, most studies reported increases in throwing velocity of around 3% [18, 20, 22–24], while two studies reported increases of around 11% [15, 19]. Hermassi et al. [21] reported increases of 16.7–24.2% in the different throws. Only Ortega-Becerra et al. [16] reported no increase in velocity after training with underweight balls. A reason that Ortega-Becerra et al. [16] did not find any positive effect, while van den Tillaar [3] found in his review that training with underweight balls always increases throwing velocity, could be the size and weight of the ball (tennis ball). Training with tennis balls is probably too removed in terms of coordination from throwing a regular handball to make transfer possible. The large increases in throwing velocity in Skoufas et al. [19] and Ekaterini et al. [15] were probably caused by the level of the subjects (novices). Thereby, a large part would be a general learning effect, as the control group, which threw the same number of throws, also had an increase of around 6.3%. Hermassi et al. [37] found very large increases in throwing velocity in the different throws (standing, running, and jump shot) in elite handball players during the season after throwing with a 3-kg medicine ball in training. This was much more effective than throwing training using regular balls. However, as mentioned before, the increases were much greater than in most other studies, and the control group that threw with the same workload (with regular handballs) also increased 16% in the jump shot. The absolute difference between the two groups was just + 1.2 m/s, which was more in line with the other underweight and overweight studies (Table 1). Reasons for these high increases could be the inexperience in the test situation or test bias, as in previous studies with medicine balls in overhead throwing increases of only around 3–5% were found after an intervention period with throwing 3-kg medicine balls [39, 40].

Only two studies [15, 17] were found that used forearm loading. Both found increases of around 13%, which is rather high. This was mainly caused by the fact that the subjects were novices in handball, and thus a large part would be a general learning effect from just an increased number of throws, as the control group that threw the same number of throws also had an increase of around 6.2%. Therefore, only a real increase between groups of 5.3% was found, as Kotzamanidis et al. [17] reported. However, both forearm loading studies [15, 17] were performed at the same institute and had almost the same increases, the same training, and the same number of subjects, which could indicate that the studies were actually just only one study. Since Ekaterini et al. [15] also had an underweight ball training group that had the same protocol, number of subjects, and increase in velocity in the control groups (+ 1.1 m/s) as Skoufas et al. [19] had in their study, it seems that the study by Ekaterini et al. [15] may have been divided in two and again reported by Skoufas et al. [19] and Kotzamanidis et al. [17]. Thus, more studies in this category, by other research groups, should be conducted to confirm if this type of training has a positive effect on throwing velocity.

After reviewing all these studies, there are no clear answers for which specific training method gives the most positive increase in throwing velocity performance, because of conflicting results in the different categories, a lack of significant differences versus control groups, and several shortcomings of the reviewed studies. A limitation of our review is that generalisation of our findings may be limited to the specific protocol applied in the reviewed studies and for that population. Furthermore, there are not enough studies in some of the categories (pulley device and forearm loading), meaning future studies should investigate these training methods so that a clear statement can be made about these methods. Furthermore, most of the studies have involved men, and more studies should include females.

## Conclusion

Based on the findings of the present review, no clear conclusion can be made on which of the specific strength training methods is best for increasing throwing velocity. However, some recommendations can be offered based on our findings. Firstly, the throwing training period should be long enough (≥ 6 weeks) with a high enough workload. Throwing training with a resistance band increases throwing velocity significantly for junior and less experienced overarm-throwing athletes based on our findings. Furthermore, throwing with underweighted balls of similar size will ensure a positive effect on throwing velocity, which agrees with earlier findings [3]. Also, throwing training with combined over- and underweighted balls can be used if the overweighted balls are carefully selected to ensure there is no negative impact on throwing kinematics and injuries. The neural adaptation and increased strength of the involved muscles can explain the increase in these training studies. Exercise intensity and coordination are also important factors and must be controlled for in the training to avoid injuries.

## Data Availability

Please contact the corresponding author for data requests.

## Abbreviations

F: Female
JT: Jump shot
M: Male
RT: Running shot
ST: Standing throw
Wk: Week
